# The Emerging Roles of Exosomes as EMT Regulators in Cancer

**DOI:** 10.3390/cells9040861

**Published:** 2020-04-02

**Authors:** Hyunwoo Kim, Sungmin Lee, Eunguk Shin, Ki Moon Seong, Young Woo Jin, HyeSook Youn, BuHyun Youn

**Affiliations:** 1 Department of Integrated Biological Science, Pusan National University, Busan 46241, Korea; harlemkim@gmail.com (H.K.); smlee1048@gmail.com (S.L.); egshin94@gmail.com (E.S.); 2 Laboratory of Low Dose Risk Assessment, National Radiation Emergency Medical Center, Korea Institute of Radiological & Medical Sciences, Seoul 01812, Korea; skmhanul@kirams.re.kr (K.M.S.); ywjin@kirams.re.kr (Y.W.J.); 3 Department of Integrative Bioscience and Biotechnology, Sejong University, Seoul 05006, Korea; 4 Department of Biological Sciences, Pusan National University, Busan 46241, Korea

**Keywords:** Epithelial mesenchymal transition, Exosome, Cancer, Wnt/β-catenin pathway, Hippo pathway

## Abstract

Epithelial–mesenchymal transition (EMT) causes epithelial cells to lose their polarity and adhesion property, and endows them with migratory and invasive properties to enable them to become mesenchymal stem cells. EMT occurs throughout embryonic development, during wound healing, and in various pathological processes, including tumor progression. Considerable research in the last few decades has revealed that EMT is invariably related to tumor aggressiveness and metastasis. Apart from the interactions between numerous intracellular signaling pathways known to regulate EMT, extracellular modulators in the tumor microenvironment also influence tumor cells to undergo EMT, with extracellular vesicles (EVs) receiving increasing attention as EMT inducers. EVs comprise exosomes and microvesicles that carry proteins, nucleic acids, lipids, and other small molecules to stimulate EMT in cells. Among EVs, exosomes have been investigated in many studies, and their role has been found to be significant with respect to regulating intercellular communications. In this review, we summarize recent studies on exosomes and their cargoes that induce cancer-associated EMT. Furthermore, we describe the possible applications of exosomes as promising therapeutic strategies.

## 1. Introduction

Epithelial–mesenchymal transition (EMT) is a reversible cellular process that transforms epithelial cells into quasi-mesenchymal cell states. It is observed during normal development, embryonic development, wound healing, and certain pathological processes. During EMT, cells lose their adhesion property, apical–basal polarity, and basement anchoring, and gain migratory and invasive properties. In tumor cells, EMT induces invasiveness and metastatic properties that can lead to malignancy, resulting in poor prognosis for the patient [1]. Additionally, EMT often induces the development of therapeutic resistance through acquisition of stemness and inhibition of tumor cell death [2,3,4]. Although several studies have investigated the suppression of EMT in tumor cells, the underlying mechanisms have not yet been fully elucidated. Many studies have identified the intracellular signaling pathways involved in EMT and their corresponding regulators. Several tumor-cell-derived growth factors, such as transforming growth factor β (TGF-β), hepatocyte growth factor (HGF), epidermal growth factor (EGF), fibroblast growth factor (FGF), Wnt ligand, interleukin-6 (IL-6), and hedgehogs act as extracellular EMT inducers and activate downstream signaling, including the TGF-β/SMAD, PI3K/ protein kinase B (AKT), mitogen-activated protein kinases (MAPKs), and Wnt/β-catenin signaling pathways [5]. Although downstream transcription factors including β-catenin, Zinc finger protein SNAI1 (SNAIL)/Zinc Finger Protein SNAI2 (SLUG), Zinc finger E-box-binding homeobox 1/2 (ZEB1/2), and Twist-related protein 1 (TWIST) govern EMT-related gene expression, recent studies suggest that controlling the expression and function of these factors does not completely inhibit EMT induction in tumor cells [6,7,8].

Recently, extracellular vesicles (EVs) have been identified as important communicators between tumor cells and their microenvironment. EVs are lipid-bilayer-delimited particles that are released from cells, and are usually categorized into at least three classes: exosomes, microvesicles, and apoptotic bodies. They carry proteins, nucleic acids, lipids, metabolites, and even organelles from donor cells. EVs enter recipient cells via endocytosis and release their bioactive contents into the cytosolic space. Among the three types of EVs, previous studies have found that cancer-derived exosomes considerably induce EMT in recipient cells, and identified the major molecules contained in the exosomes [9,10,11]. Furthermore, it has been reported that regulation of exosomal bioactive molecules such as nucleotides, proteins, and organic intermediates can be employed as a strategy to suppress EMT.

In this review, we summarize the EMT-regulating molecules contained in exosomes and describe the associated molecular mechanisms. In addition, we suggest that exosomal contents can potentially be used for predicting tumor prognosis and in the formulation of personalized therapies to overcome tumor EMT induction.

## 2. Exosome-Induced EMT

**Figure 1:**
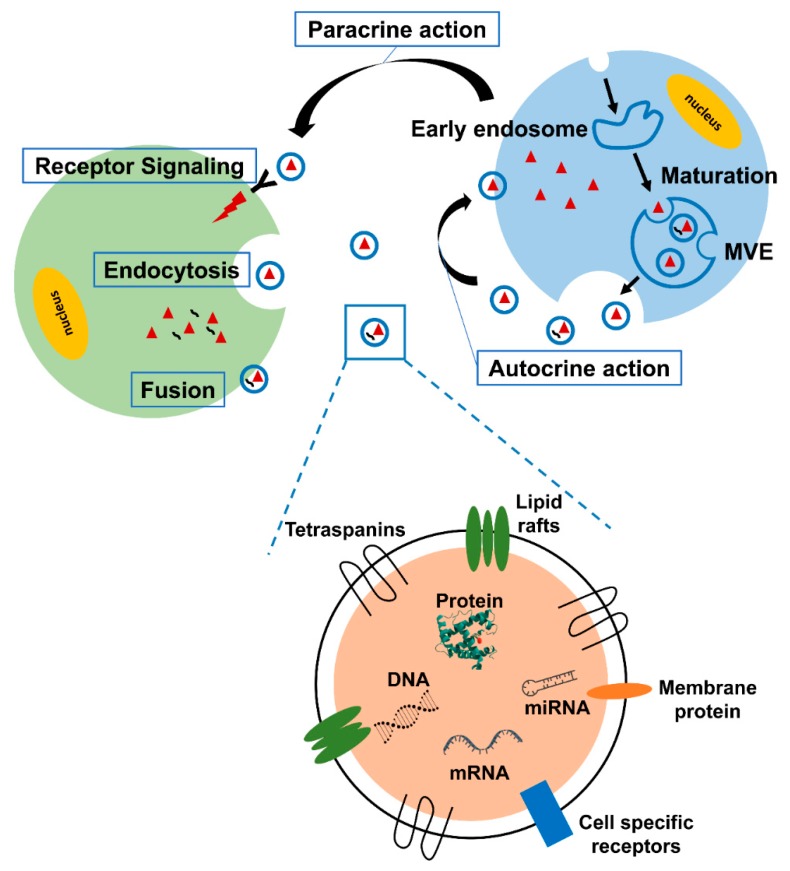
Schematic representation of exosomes. Exosomes are generated from multivesicular endosome (MVE), loaded with cellular components in donor cells, and secreted to the extracellular environment. Exosomes transfer proteins, nucleic acids, metabolites, and even organelles to recipient cells in both autocrinal and paracrinal manners. The uptake of exosomes is mediated by receptor activation, endocytosis, and membrane fusion.

Exosomes are secretory endosome-derived membranous vesicles with a size of ~40–100 nm, secreted from a various types of mammalian cells. The term “exosome” was first coined by Trams et al. in the early 1980s. Exosomes were initially assumed to be garbage disposal agents; however, recent data have revealed the direct role of exosomes in governing the physiological and pathological conditions of the surrounding cells by transferring information from donor cells to recipient cells (Figure 1). The generation of exosomes begins from endocytosis to form early endosomes and multivesicular endosomes (MVEs) are formed by inward budding of late endosomes. The MVEs merge with the cell membrane and release the intraluminal endosomal vesicles to become exosomes into the extracellular space [12,13]. Exosome can transport all the biomolecules, including lipids, proteins, DNAs, mRNAs, and miRNA [14]. Most of the contents in exosomes are proteins related with biogenesis and transportation ability of exosomes. Tetraspanins (CD9, CD63, CD81, CD82) take charge of cell penetration, invasion, and fusion events. MVB formation proteins (Alix, TSG101) are involved in exosome release. Annexins and Rab protein have roles in membrane transport and fusion. Alix, fotillin, and TSG101 are participants in exosome biogenesis [15,16]. These exosome abundant proteins (TSG101, HSP70, CD81, CD63) are commonly used as exosomal marker proteins [17]. Besides, other exosomal contents involved in the intercellular crosstalk in the tumor microenvironment plays a large role in cancer development through the formation of a mesenchymal niche. Recently, many studies have been conducted to investigate the significant involvement of exosomes in the induction of cancer-related EMT. We summarized these reports and proposed that the critical components in exosomes can serve as promising EMT regulators, based on the signaling pathways and exosome-derived materials involved.

**Figure 2:**
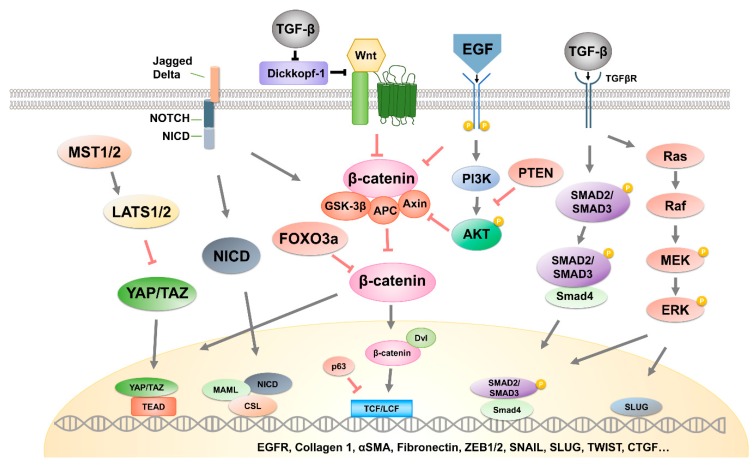
Comprehensive schematic diagram of signaling pathways related to exosome-induced EMT in cancer. The principal signaling pathways and transcription factors involved in exosome-mediated EMT induction are depicted.

In the next section, we describe the major exosomal cargo molecules (oligonucleotides, proteins, lipids, etc.) that cause the activation of EMT-related signaling pathways (Figure 2).

### 2.1. Hippo Pathway

Numerous studies have shown that the Hippo tumor suppressor signaling pathway has a crucial role in restricting organ size by inhibiting the oncogenic co-activators Yes-associated protein (YAP)/transcriptional coactivator with PDZ-binding motif (TAZ) [18,19,20]. Upon inactivation of the Hippo pathway, unphosphorylated YAP/TAZ translocates to the nucleus and bind to the TEA Domain Transcription Factor (TEAD) transcription factor [21,22]. TEAD-mediated target gene transcription increases the expression of mesenchymal markers such as Vimentin and N-cadherin, while simultaneously inhibiting the expression of epithelial markers including E-cadherin [23,24,25,26]. Additionally, YAP interacts with EMT-induced transcription factors ZEB1, SNAIL, and SLUG, and their complex promotes cancer stem cell (CSC) traits [27,28,29]. Several recent studies have revealed that exosomes could induce EMT through modulation of the Hippo pathway. Exosomes from mesenchymal-stem-cell-derived adipocytes promoted Hippo-mediated EMT in breast cancer cells, while their principal cargo was not identified [30]. In hepatocellular carcinoma cells, exosomal miR-665 has been suggested to serve as a novel invasive biomarker [31]. A previously conducted mechanistic study showed that miR-665 directly suppressed tyrosine phosphatase receptor type B (PTPRB) expression as well as Hippo-mediated EMT in tumor cells [32]. In addition to exosomal microRNAs, chronic-myelogenous-leukemia-derived exosomal amphiregulin (AREG) activated Epidermal growth factor receptor (EGFR) and SNAIL expression [33]. AREG has been reported to be involved in the formation of a feedforward cycle of EGFR/TAZ/AREG and in the induction of EMT [34]. These results suggest that exosomal miRNA and proteins targeting the Hippo pathway could induce EMT in recipient cells.

### 2.2. β-Catenin Signaling Pathway

The Wnt/β-catenin signaling pathway is one of the most widely studied of the pathways that trigger EMT, and β-catenin is a key molecule involved in cancer-promoting pathways [35,36]. This signaling pathway also activates several EMT-related transcription factors, such as SNAIL, SLUG, and TWIST [37,38]. In studies about exosomes, Wnt/β-catenin signaling pathway is one of the most common target pathways.

Exosomal miR-301a is secreted by hypoxic glioblastoma (GBM) cells and targets transcription elongation factor A like 7 (TCEAL7), which, in turn, results in the activation of the Wnt/β-catenin signaling pathway [39]. In a previous study, miR-301a was found to induce EMT in prostate cancer cells through inhibition of p63, which caused the release of ZEB1/2 in a miRNA-mediated inhibition and induction EMT [40]. Exosomal miR-1260b reportedly activates the Wnt/β-catenin signaling pathway in lung adenocarcinoma cells [41]. miR-1260b was suggested to directly suppress suppressor of cytokine singling 6 (SOCS6) expression and activate KIT proto-oncogene (KIT) signaling in non-small-cell lung cancer (NSCLC) [42]. KIT signaling is known to induce EMT via the KIT-MEK-ERK pathways, supporting the significant roles of miR-1260b [43]. In another study, it was reported that miR-92a in fibroblast-derived exosomes activates the Wnt/β-catenin signaling pathway and induces EMT in colorectal cancer cells [44]. Additionally, miR-92a induced EMT by regulating the PI3K/AKT signaling pathway by targeting PTEN in NSCLC [45]. Similarly, exosomal delivery of miR-155 promoted EMT in breast cancer cells [46]. Although the underlying molecular mechanisms were not investigated in the study, miR-155 has been suggested to be oncogenic and EMT-inducible [47,48,49]. In the context of EMT, miR-155 activated the PI3K/SGK3/β-catenin signaling pathways through direct regulation of p85α expression [50,51]. Lastly, overexpressed *PRMT5* circular RNA (circPRMT5), secreted via exosomes was found to promote EMT in recipient cells both in vivo and in vitro via sponging miR-30c [52,53]. Another similar study found that miR-30c targeted BCL9, a coactivator of the Wnt/β-catenin signaling pathway [54]. In a similar study, circABCC1 present within colorectal-cancer-cell-derived exosomes reportedly induced Wnt/β-catenin signaling activation in recipient cells, but the underlying molecular mechanisms were not investigated [55].

In studies about exosome-mediated Wnt/β-catenin signaling pathway, many Wnt ligands have been shown to be delivered via exosomes for biological function. Exosomal delivery of Wnt1 induced migration of colorectal cancer cells through activation of Wnt signaling, which was suppressed by inhibition of exosome production [56]. Wnt3a in fibroblast-derived exosomes induced activation of Wnt signaling and the development of drug resistance in colorectal cancer cells [57]. Wnt5a in exosomes from endothelial cells was biologically active and increased pericyte recruitment [58]. Wnt10b delivered by exosomes from fibroblasts induced EMT in breast cancer cells [59]. Fibroblast-derived exosomes promoted autocrine Wnt11a stimulation through formation of the PCP complex that is essential for exosome-mediated EMT induction in cancer cells [60,61]. Hypoxic-colorectal-cancer-cell-derived exosomal Wnt4 activated the β-catenin signaling pathway in endothelial cells, which contributed to colorectal cancer progression [62]. In large B-cell lymphoma, exosome-mediated Wnt3a activated the Wnt signaling pathway and promoted the progression of lymphoma cells [63]. Colorectal-cancer-cell-derived exosomes containing mutant β-catenin activated Wnt signaling in recipient cells in an autocrine manner [64]. Macrophage-derived exosomes containing Wnt5a activated the Wnt/β-catenin signaling pathway in breast cancer cells; this was further supported by the fact that Wnt5a increased exosome production in melanoma cells [65,66]. Therefore, regulation of Wnt ligands present in exosomes could serve as a promising therapeutic strategy to control EMT induction in cancer cells.

Significant roles of exosome-derived proteins have also been reported in the context of Wnt/β-catenin pathway activation. A recent study found that the EMT regulator SNAIL moves directly to the exosomes. Exosomes released by cancer-associated fibroblasts (CAFs) transferred SNAIL to lung cancer cells, and this SNAIL induced EMT in recipient lung cancer cells [67]. Deficiency of p53 specifically increased the expression of ubiquitin regulatory protein ligase E3 component N-recognin 2 (UBR2) in bone marrow mesenchymal stem cells as well as in its exosomes, activating Wnt signaling and downstream gene expression in gastric cancer cells [68]. Recently, a large extracellular matrix glycoprotein, Tenascin-C (TNC), was identified as an exosomal protein capable of inducing EMT. TNC is transferred by autocrinal exosomes from pancreatic ductal adenocarcinoma cells, and was found to promote EMT in the pancreatic ductal adenocarcinoma (PDAC) cell line via regulation of the Wnt/β-catenin pathway [69]. In breast cancer cells, TNC induces EMT by activation of SRC proto-oncogene tyrosine kinase (SRC) through phosphorylation at Y418 and through phosphorylation of focal adhesion kinase (FAK) at SRC substrate sites Y861 and Y925 [70].

Analysis of the expression of SRY-Box transcription factor 2 (SOX2) overlapping transcript (Sox2ot)—a long non-coding RNA (lncRNA)—derived from exosomes of highly invasive PDAC cells in plasma samples revealed that the plasma exosomal Sox2ot expression was high and correlated with TNM classification of malignant tumor stage and overall survival rate of PDAC patients. Further research showed that Sox2ot promotes EMT and stem-cell-like properties by regulating Sox2 expression. Sox2ot competitively binds to the miR-200 family to regulate the expression of Sox2, thus promoting invasion and metastasis of PDAC [71]. Previously, Sox2 inhibits Wnt/β-catenin signaling pathway through phosphorylation of β-catenin and glycogen synthase kinase 3 beta (GSK3β), leading to inhibition of the downstream gene expression [72]. The transfer and expression of long intergenic non-coding RNA regulator of reprogramming (linc-ROR) by CSC exosomes is responsible for the induction of EMT and the significant increase in the invasive potential of normal thyroid cells. Exosomal transfer of linc-ROR, a long non-coding RNA, was reported to induce EMT through the formation of a distant metastatic niche [73]. The significant role of linc-ROR through the Wnt/β-catenin pathway has been previously suggested in various types of cancers [74,75]. Furthermore, linc-ROR sponged miR-205 and increased the stability of the EMT inducer, ZEB2 [74]. These studies suggest that exosomal delivery of lncRNA may cause EMT induction.

### 2.3. ERK Pathway

miR-21 shows features of an oncogenic miRNA by targeting many tumor suppressor genes related to cell proliferation, apoptosis, and invasion in multiple histological cancer types, including OSCC. Hypoxia enhanced miR-21 levels in both OSCC cells and OSCC-derived exosomes. This induction of miR-21 by hypoxia was directly regulated by HIF-1a and HIF-2a in OSCC cells [76]. Additionally, inhibition of miR-21 reversed EMT and CSC phenotype, accompanied by phosphatase and tensin homolog (PTEN) upregulation and AKT/extracellular Signal Regulated Kinase (ERK)1/2 inactivation. Interestingly, downregulation of PTEN by siPTEN suppressed the effects of miR-21 antagomir on EMT and CSC phenotype, confirming that PTEN is a target of miR-21 during reversal of EMT and CSC phenotype in breast cancer cells [77].

Studies on liver cancer suggest that controlling exosome production and secretion could regulate exosomal EMT induction. Rab27a, a small GTPase, is required for efficient secretion of exosomes via mediation of multivesicular endosome docking at the plasma membrane [78]. In HLE (hepatoma cell line), the MAPK/ERK-mediated EMT was induced by MHCC97H hepatoma-cell-derived exosomes, and Rab27a knockdown reduced exosome secretion as well as EMT induction. Exosomes released by gastric cancer cells enhanced tumor proliferation by activating the MAPK/ERK pathway [79]. A similar result was obtained in a study that demonstrated that exosomes from hepatocellular carcinoma (HCC) cells increased ERK activity in normal hepatocytes [80].

An exosomal lncRNA, HOTAIR (Hox antisense intergenic RNA), was found to be highly expressed in bladder cancer patients and induced the invasiveness of the tumor through induction of EMT [81,82,83,84]. HOTAIR bound to miR-29b and rescued expression of DNMT3b, leading to PTEN expression, activation of the ERK and AKT signaling, and activation of EMT signaling pathway [85].

## 3. Therapeutic Candidates for Preventing Exosomal EMT Induction

Based on the roles of exosomes with respect to transferring bioactive molecules from donor cells to recipient cells, some investigators noticed that delivery of tumor-suppressive molecules via exosomes may regulate tumor progression. This was in accordance with some reports suggesting the significant involvement of exosomal delivery of nucleic acids in the inhibition of EMT in tumor cells, and their potential for therapeutic application. In this section, we summarize promising exosomal contents that prevent EMT in recipient tumor cells and their molecular relevance, based on previous studies.

In a study performed using cholangiocarcinoma cells, it was confirmed that exosome-encapsulated miR-30e delivery could suppress EMT in tumor cells through inhibition of SNAIL expression [86]. miR-30e has been widely studied and suggested to be a tumor suppressive miRNA that regulates tumor growth, invasion, and metastasis [87,88]. In the context of EMT induction, miR-30e is an important EMT inhibitor that works by targeting ITGB1, TUSC3, USP22, and SOX9 mRNAs, which reportedly induce EMT by activating transcription factors including SNAIL, SLUG, TWIST, and ZEB1/2 [89,90,91,92]. The preclinical significance of miR-30e treatment for EMT regulation was validated in tumor xenograft models, making exosomal miR-30e delivery a promising therapeutic strategy.

Recently, the EMT-suppressive effects of exosomal miR-26a were demonstrated in prostate cancer cells [93]. Although specific molecular events governed by miR-26 were not fully described, miR-26a was reportedly downregulated in tumor tissues and prevented EMT through direct inhibition of connective tissue growth factor (CTGF), rho-associated, coiled-coil-containing protein kinase 1 (ROCK1), enhancer of zeste homolog 2 (EZH2), and Jagged-1, which led to inactivation of the Jagged-1/Notch and TGFβ1/SMAD pathways [94,95,96,97,98]. Among the target genes, *CTGF* and *ROCK1* indicated the involvement of miR-26a in YAP/TAZ signaling and further emphasized the importance of the miRNA [99].

miR-34a was supposed to be one of the foremost tumor-suppressive miRNAs, and the molecular mechanisms underlying its function were extensively studied [100]. A study on fibroblast-derived exosomes revealed that the level of miR-34a was significantly reduced in exosomes from CAFs, and that overexpression of miR-34a through exosomal transfer could suppress EMT in oral squamous cancer cells [101]. As miR-34a also reportedly targets signal transducer and activator of transcription 3 (STAT3), lymphoid Enhancer Binding Factor 1 (LEF1), ZEB1, and SNAIL, direct regulation of EMT could be achieved by treatment with miR-34a [102,103,104,105].

Exosomal miR-34c, a functionally similar family member of miR-34a, was reported to inhibit EMT by targeting β-catenin in nasopharyngeal cancer cells. Tumor-suppressive miR-34c was often downregulated in various types of tumor cells and regulated EMT through direct binding with SOX9, SATB2, MAP3K2, and DANCR mRNA [106,107,108,109,110]. The clinical prospects of miR-34c were substantiated by a study which showed that miR-34c inhibited exosome shedding by directly targeting RAB27B, thereby terminating the vicious cycle of oncogenic exosome-mediated intercellular communication [111].

A recent study about exosomes from bone-marrow-derived mesenchymal stem cells found that exosomal miR-101 inhibited EMT in oral cancer cells [112]. Although the molecular mechanisms were only elucidated to regulate COL10A1 expression, miR-101 reportedly played a role in EMT inhibition through direct inhibition of ZEB1/2, SOX9, TRIM44, ROCK2, and DUSP1, which supports its functional relevance [113,114,115,116,117].

Exosomes from miR-143 overexpressing bone-marrow-derived mesenchymal stem cells suppressed EMT in prostate cancer cells by directly targeting TFF3 mRNA [118]. Although not much is known about the role of TFF3 in EMT regulation, its significant correlation with TWIST1 has been reported in colorectal cancer [119]. The relationship between miR-143/TFF/EMT was also supported by previous studies which showed that miR-143 directly targets MYO6, HMB1, ERK5, Activin A, and LAMP3 mRNA, and eventually inactivates TWIST1, SNAIL, and SLUG [120,121,122,123,124].

Although the roles of miR-148b in regulation of tumor repression have been widely studied, only a few studies suggested ROCK1 and BRG1 as molecular targets related to EMT regulation [125,126]. Nevertheless, two studies have suggested that miR-148b in exosomes derived from mesenchymal stem cells and fibroblasts suppress EMT in recipient tumor cells [127,128]. Further studies are warranted to elucidate the molecular mechanisms underlying the function of exosomal miR-148b in EMT regulation.

In a previous study, exosomal miR-200 family reduced the invasiveness of colon cancer through inhibition of ZEB1 and SLUG [129]. With respect to the molecular mechanisms, the miR-200 family is known to be significantly involved in the induction of EMT via targeting of ZEB1, NANOG, and FOXF2 mRNA [130,131,132]. Among them, the effects of ZEB1 expression were clearly evident in a couple of studies. However, another study reported that extracellular vesicles containing miR-200 promoted cancer metastasis in xenograft mouse models [133]. As the EMT-suppressive roles of miR-200 have been studied widely, further studies could clarify the precise roles of the exosomal miR-200 in EMT regulation.

The application of EMT-suppressive miR-375 was ascertained in a study that found that treatment with *Emblica officinalis* extracts causes exosomal miR-375 to increasingly target SNAIL and inhibit invasiveness of ovarian cancer [134]. Similarly, exosomal miR-375 suppressed EMT in glioblastoma cells through direct targeting of SLC31A1 mRNA [135]. miR-375 is often downregulated in various types of cancers and shows tumor-suppressive effects by directly targeting various oncogenes at the mRNA level [136]. With respect to EMT induction, the regulation of YAP1 expression by direct binding of miR-375 was controlled by ZEB1 in prostate cancer, thereby forming a feedforward cycle [137]. Additionally, miR-375 inhibited the expression of 14-3-3ζ, which led to inhibition of the Wnt/β-catenin signaling pathway and EMT in gastric cancer [138]. These studies demonstrated the significant involvement of miR-375 in regulation of EMT in cancer cells.

These studies cumulatively proved that miRNAs previously known to regulate EMT also showed the same effects upon exosomal delivery, the exosomes being obtained from mesenchymal stem cells with transfection of the desired miRNAs. It can be theorized that a universal recipe for exosomal encapsulation of miRNAs may be developed, wherein these constructs serve as adjuvants for anti-cancer therapy based on these investigations. Although many studies have established the preclinical significance of co-treatment of miRNAs with anti-cancer therapies including chemotherapy and radiotherapy, the therapeutic effects of combining multiple miRNAs have not been widely investigated. For better therapeutic significance, a combination of several miRNAs mentioned above could be formulated to achieve synergistic effects on the regulation of tumor EMT and progression in future studies.

## 4. Clinical Application

To date, many studies have been conducted to understand the biological functions of exosomes, and some have identified clinical utility. Most clinical studies have used exosomes as biomarkers for diagnosis or prognosis of disorders including cancers. In the same context, exosomes containing EMT inducers have been utilized for prediction of metastatic prognosis of cancers in clinical trials. For example, profiling exosomal RNA in primary high-grade osteosarcoma metastasizing to the lung could provide diagnostic and prognostic biomarkers [139] (NCT03108677). In addition, one investigation analyzed circulating exosomes and miRNAs in cancer patients to predict the possibility of bone metastasis [140] (NCT03895216). Additionally, exosomes were recently utilized as a treatment. A vaccination of tumor antigen was mediated by dendritic-cell-derived exosomes [141] (NCT01159288), and a transfer of curcumin to normal and cancerous colon cells was mediated by plant exosomes [142] (NCT01294072). In clinical trials with pancreatic cancer patients, attempts were made to target the mutant form of the GTPase KRAS (KRASG12D) by transfer of siRNA mediated by mesenchymal-stromal-cell-derived exosomes (iExosomes) [143,144] (NCT03608631). Although there have not been many clinical investigations performed to date, we present in this section studies that confirmed therapeutic effects and show a marked potential for clinical applications.

As described above, miRNAs and proteins delivered via exosomes have exhibited potential as EMT regulators in a variety of cancers. As most studies were performed using cell models, data regarding studies performed on in vivo models are insufficient. There are two representative types of therapeutic strategy for inhibiting exosome-induced EMT. One is the transfer of tumor-suppressive factors through exosomes, and the other is the inhibition of EV production. This section discusses previous studies covering the in vivo effects of exosomes in regulating EMT in tumors.

There have been many attempts to incorporate various compounds into exosome therapy. Exosomes from the plasma of head and neck squamous cell carcinoma (HNSCC) patients treated with photodynamic therapy (PDT) downregulated the expression of SLUG and ZEB1 [145]. Studies on cancer EMT revealed that transcriptional activation of ZEB1 by SLUG promoted EMT progression that decreased adhesion and increased migration of cancer cells [146]. Exosomes from patient plasma could be used for management of EMT as part of a novel therapeutic approach. In a previous study, exosomes packed with an miR-21-sponge were successfully employed in a GBM rat model, suggesting that this construct could have EMT-inhibitory effects [147]. miR-21 targets several EMT pathway-related genes, such as *PDCD4*, *TIMP3*, and *RECK* [148]. This strategy of inhibition using exosomes containing an miR-21-sponge construct has therapeutic potential for blocking GBM and GBM-induced EMT. Another study suggested that suppressing miR-21 also enhanced the therapeutic effects of sunitinib-based chemotherapy [149].

Another approach is therapy based on the use of exosomes that repress tumor metastasis by targeting EMT inducers. The findings of studies on this therapy have been difficult to apply in clinical trials, but these studies might contribute toward developing a therapeutic strategy against cancer-induced EMT.

In a study on mouse- or rat-derived exosomes, artificial exosomes loaded with miRNAs targeting Claudin 7 and EpCAM through electroporation were intravenously injected, resulting in significant inhibition of cancer EMT and metastasis [150]. Claudin 7 promotes EMT in human colorectal cancer and is required for EpCAM expression [151]. For therapeutic applications, the use of exosomes has beneficial effects in the management of EMT. In a recent study, EVs obtained from bone marrow mesenchymal stem cells overexpressing miR-200b prevented EMT by targeting ZEB1 and ZEB2 mRNA. Although the therapeutic target was not cancer cells, the result suggested a significant role of miR-200b in clinical applications for regulation of EMT [152].

Another study demonstrated that the tumor suppressive miR-335 could be produced in B cells by plasmid DNA induction (iEVs) which is suggested a new therapeutic method utilizing exosomes and showed anticancer effects. miR-335 has been reported to inhibit tumor re-initiation, and is known to be widely silenced in genetic and epigenetic manners during tumorigenesis. Induced exosomal miR-355 can overcome this limitation. One of the targets of miR-335 is SOX4, a transcription factor involved in embryonic development, cell fate determination, and EMT [153].

Although there have been few clinical attempts to inhibit exosome production in order to regulate the cancer EMT, there has been a report supporting its anti-tumorigenic roles. A study prevented EV production using cetuximab, a monoclonal antibody targeting EGFR, and was successful in reducing the production of EMT-inducing exosomes from oral cancer cells [154].

In addition to the two major therapeutic strategies described above, some studies have suggested new approaches that need to be improved for their clinical application. One group posited exosomes or exosome-like platforms for anticancer drug delivery. Exosomes derived from brain endothelial cells overexpressing CD63 tetraspanin transmembrane proteins successfully penetrated the blood-brain barrier (BBB) and delivered containing anticancer drugs in the zebrafish brain cancer model. Exosome-delivered drugs significantly decreased the growth of xenografted cancer cells [155]. Another group devised exosome-mimetic nanosystems (EMNs) that simulated natural tumor-derived exosomes with respect to their structure and functionality, but with a controlled composition, for targeted delivery of therapeutic oligonucleotides to lung adenocarcinoma cells. These EMNs delivered miRNA-145 to lung adenocarcinoma cells [156]. These studies offer new strategies for the delivery of anti-cancer, anti-EMT drugs, and therapeutic oligonucleotides via artificially exosomes or exosome mimics. Further research could open avenues to new anticancer therapies by delivering drugs and miRNAs that are effective against a variety of cancers or a variety of tumorigenic processes, including EMT.

## 5. Conclusions

**Figure 3:**
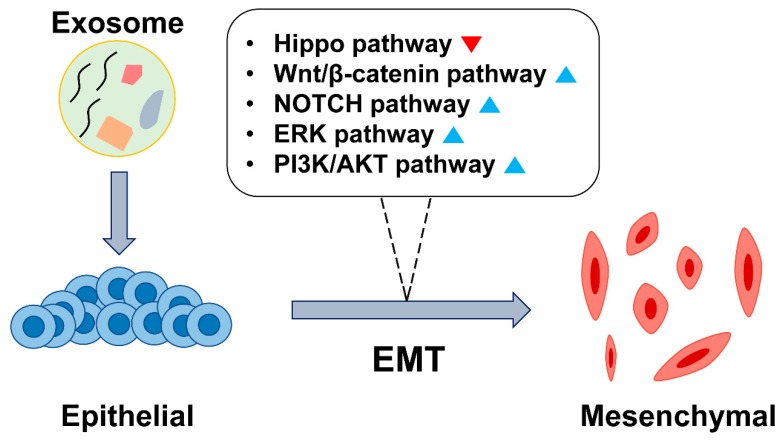
Schematic representation of exosome-induced EMT in cancer. The involvement of EMT-related signaling pathways during exosome-mediated EMT is depicted. The blue triangle indicates activation and red inverted triangle indicates inactivation of the signal.

In this review, we summarized the major exosomal molecules that induce EMT and their underlying molecular mechanisms (Figure 3, Table 1). Previously known EMT-related signaling pathways are involved in exosome-induced EMT, miRNAs being the important mediators. Furthermore, we reviewed studies on therapeutic candidates that can be delivered via exosomes and their clinical applications as anti-cancer therapies. Although there is more to be discovered about the roles of exosomes, it is undeniable that exosomes play a critical role in the regulation of EMT in recipient tumor cells. This research review should be helpful in providing a deeper understanding of exosome-induced EMT, and could also guide researchers pursuing novel investigations in this area.

## Figures and Tables

**Table 1:** The contents of exosomes and their mechanisms leading to EMT in cancer.

Name		Cancer Type	Target	Signaling Pathway	Reference
miRNA	miR-665	Hepatocellular carcinoma	PTPRB	Hippo pathway	[23,24]
	miR-34c	Nasopharyngeal carcinoma	β-catenin, SOX9, SATB2, MAP3K2, DANCR, RAB27B	Wnt/β-catenin pathway	[92,93,94,95,96,97]
	miR-301a	GBM	TCEAL7, p63, ZEB1, ZEB2, E-cadherin		[31,32]
	miR-92a	Colorectal cancer	FBXW7, MOAP1		[36]
	miR-155	Breast cancer	FOXO3a, SGK3, p85a		[38,39,40,41,42,43]
	miR-30c	Esophageal cancer	circPRMT5, BCL9		[44,45,46]
	miR-1260b	Lung adenocarcinoma	SOCS6, YY1		[33]
	miR-375	Gastric cancer	14-3-3ζ, YAP, SNAI1, SLC31A1		[120,121,122,123,124]
	miR-34a	OSCC	STAT3, LEF1, ZEB1, SNAI1	NOTCH signaling pathway	[87,88,89,90,91]
	miR-1260b	NSCLC	KIT, MEK, ERK	ERK pathway	[34,35]
	miR-21	OSCC	HIF-1a, HIF-2a, PTEN		[64]
	miR-92a	NSCLC	PTEN, PI3K, AKT	PI3K/AKT pathway	[37]
Protein	AREG	Chronic myelogenous leukemia	EGFR, SNAI1, YAP/TAZ	Hippo pathway	[25,26]
	linc-ROR	Breast cancer	miRNP, miR-205, ZEB2	Wnt/β-catenin pathway	[69]
	Cir-ABCC1	Colorectal cancer	Unclear		[47]
	UBR2	Gastric cancer	p53		[60]
	TNC	PDAC	E-cadherin, CD44, MMP-7, MET		[61]
	TNC	Breast cancer	p-Y418, p-FAK, SRC		[62]
	Wnt1	Colorectal cancer	TCF/LEF		[48]
	Wnt3a	Colorectal cancer	TCF/LEF		[49]
	Wnt4	Colorectal cancer	HIF1α		[54]
	Wnt5a	Breast cancer	p38		[50,57,58]
	Wnt10b	Breast cancer	p85a		[51]
	Wnt11a	Breast cancer	PCP		[52,53]
	HOTAIR	Bladder cancer	PTEN, miR-29b, DNMT3b	MAPK/ERK pathway	[81,82,83,84,85]
	Cbl	Liver cancerGastric cancer	Rab27a, AKT		[65,66,67]
	lncRNA-Sox2ot	PDAC	Sox2ot, miR-200	Sox2ot pathway	[68]

## Abbreviations

ABCC1	ATP binding cassette subfamily C member 1
AKT	protein kinase B
AREG	amphiregulin
BCL9	B-cell CLL/lymphoma 9 protein
BRG1	brahma-related gene 1
CAF	cancer associated fibroblast
COL10A1	collagen type X alpha 1 chain
CSC	cancer stem cell
CTGF	connective tissue growth factor
DANCR	differentiation antagonizing non-protein coding RNA
DUSP1	dual specificity protein phosphatase 1
EGF	epidermal growth factor
EGFR	epidermal growth factor receptor
EMN	exosome-mimetic nanosystem
EMT	epithelial-mesenchymal-transition
EpCAM	epithelial cell adhesion molecule
ERK	extracellular signal regulated kinase
EV	extracellular vesicle
EZH2	enhancer of zeste homolog 2
FAK	focal adhesion kinase
FBXW7	F-Box and WD repeat domain containing 7
FGF	fibroblast growth factor
FOXF2	forkhead box F2
FOXO3a	forkhead box O3
GBM	glioblastoma multiform
HCC	hepatocellular carcinoma
HGF	hepatocyte growth factor
HIF-1α	hypoxia-inducible factor 1-alpha
HLE	human lens epithelial cell
HMB1	high mobility group box 1
HNSCC	head and neck squamous cell carcinoma
ITGB1	integrin subunit beta 1
KIT	KIT proto-oncogene
LAMP3	lysosome-associated membrane protein 3
LEF1	lymphoid enhancer-binding factor 1
MAP3K2	mitogen-activated protein kinase kinase kinase 2
MAPK	mitogen-activated protein kinase
MEK	MAP kinase-ERK kinase
MOAP1	modulator of apoptosis 1
MYO6	myosine 6
NSCLC	non-small-cell lung carcinoma
OSCC	oral squamous cell carcinoma
PCP	planar cell polarity
PDAC	pancreatic ductal adenocarcinoma
PDCD4	programmed cell death protein 4
PDT	photodynamic therapy
PI3K	phosphoinositide 3-kinase
PRMT5	protein arginine methyltransferase 5
PTEN	phosphatase and tensin homolog
PTPRB	protein tyrosine phosphatase receptor type B
RAB27B	RAB27B, member RAS oncogene family
RECK	reversion-inducing-cysteine-rich protein with kazal motifs
ROCK1	rho-associated, coiled-coil-containing protein kinase 1
ROR	RNA regulator of reprogramming
SATB2	special AT-rich sequence-binding protein 2
SGK3	serum and glucocorticoid kinase 3
SLAI1	snail family transcriptional repressor 1
SLC31A1	solute carrier family 31 member 1
SOCS6	suppressor of cytokine signaling 6
Sox2ot	SOX2 overlapping transcript
SOX9	SRY-Box transcription factor 9
SRC	SRC proto-oncogene tyrosine kinase
STAT3	signal transducer and activator of transcription 3
TAZ	transcriptional coactivator with PDZ-binding motif
TCEAL7	transcription elongation factor A like 7
TEAD	transcriptional enhanced associate domain
TFF3	trefoil factor 3
TGF-β	transforming growth factor β
TIMP3	metalloproteinase inhibitor 3
TNC	tenascin C
TRIM44	tripartite motif-containing protein 44
TUSC3	tumor suppressor candidate 3
UBR2	ubiquitin regulatory protein ligase E3 component n-recognin 2
USP22	ubiquitin specific peptidase 22
YAP	yes-associated protein
YY1	YY1 transcription factor
ZEB1/2	zinc finger E-box binding homeobox 1
